# Perioperative cognitive dysfunction in bladder cancer patients, causal factors, and possible interventions

**DOI:** 10.3389/fmed.2026.1883877

**Published:** 2026-09-03

**Authors:** Ana Cicvaric, Josipa Glavas Tahtler, Oliver Pavlovic, Celeste Dias, Ozana Katarina Tot, Anja Bjelousov Baksa, Slavica Kvolik

**Affiliations:** 1 Department of Anesthesiology, Resuscitation, and ICU, Osijek University Hospital Center, Osijek, Croatia; 2 Faculty of Medicine, Josip Juraj Strossmayer University of Osijek, Osijek, Croatia; 3 Department of Urology, Osijek University Hospital, Osijek, Croatia; 4 Neurocritical Care Unit, Medical University Center (CUME), Porto, Portugal

**Keywords:** anesthesia, bladder cancer, cognitive dysfunction, frail elderly, postoperative complications, risk factors, tobacco smoking, urinary tract infections

## Abstract

Bladder cancer (BC) primarily necessitates surgical and oncological interventions; however, patients frequently encounter a substantial burden of perioperative neurocognitive disorders, which compromise clinical outcomes and treatment compliance. While individual risks are recognized, a comprehensive synthesis identifying the overlapping clinical determinants and promoters of this decline remains essential. This review aims to elucidate the specific mechanisms underlying perioperative cognitive impairment in surgical BC patients and highlight targeted preventive strategies. Evidence demonstrates that perioperative cognitive decline in this population is highly multifactorial. Key demographic and clinical predictors include advanced age and clinical frailty, which are compounded by chronic cerebral small vessel disease secondary to long-term tobacco smoking, hypertension, and metabolic diseases. Furthermore, surgical and procedural risks, such as frequent instrumentation and catheterization, are associated with bleeding and a higher incidence of recurrent urinary tract infections, which induce systemic inflammation and may precipitate acute disorientation or delirium. Anesthesia-related factors, including preoperative benzodiazepine use, intraoperative hypotension, prolonged anesthesia duration, and the omission of neuromuscular blockade reversal agents, significantly exacerbate post-surgical cognitive vulnerability. All these factors, with systemic inflammation, may cause cerebral hypoperfusion that precipitates delirium. Metabolic comorbidities, particularly diabetes mellitus, synergistically exacerbate infection risks, while psychological distress (severe anxiety and depression) and neurotoxic chemotherapy protocols serve as vital indicators of post-surgical cognitive vulnerability. Cognitive function heavily dictates urological care trajectories. Incorporating routine, structured preoperative cognitive and frailty screenings is imperative to risk-stratify patients, implement tailored pre-habilitation, and optimize both cognitive and oncological outcomes.

## Highlights

- Preoperative cognitive dysfunction in bladder cancer patients is multifactorial.
- Early recognition of high-risk patients may guide diagnostic and therapeutic interventions, i.e., screening for preoperative cognitive impairment, smoking cessation, earlier mobilization, shorter catheterization, and detection of infections.
- The development and favoring of non-invasive diagnostics such as uTERTpm ddPCR could reduce the frequency of perioperative complications, especially when it comes to diagnostic procedures.
- A prospective study that would include these interventions could confirm whether they can improve both cognitive and oncological outcomes, i.e., disease recurrences and long-term well-being.

## 1 Introduction

Bladder cancer (BC) is among the most common tumors of the urinary system, accounting for 3.1% of all cancers globally, with a higher incidence in European countries. These patients often have numerous comorbidities and neoplastic diseases, and often undergo various diagnostic and surgical procedures. Although with improved screening, BC has recently been detected earlier and has a good prognosis, this cancer is on the rise in the EU, especially among women. Moreover, during screening, patients with non-muscle-invasive bladder cancer (NMIBC) often undergo invasive diagnostic and therapeutic procedures under anesthesia, after which numerous complications are possible (1).

Cognitive disorders are common in the perioperative period in these patients. A variety of cognitive disorders coexist with a diagnosis of BC, and advanced diseases are often associated with a greater psychological burden. Several disorders are particularly pronounced in this sensitive and turbulent period, such as cognitive decline, hypoactive and hyperactive delirium, depression, anxiety, and even suicidal ideas (2). These may be a consequence of previous diseases or risk factors that can trigger bladder cancer development, or a complication of diagnostic and therapeutic procedures performed in these patients. The aim of this article is to emphasize the importance of recognition of cognitive dysfunction in the perioperative period. After identifying patients at risk, possible interventions can be identified to improve their outcomes, while high-risk procedures may be avoided.

## 2 Incidence and burden of perioperative cognitive dysfunction

To fully appreciate the clinical impact of this issue, it is critical to first assess the baseline incidence and clinical burden of neurocognitive decline in this surgical cohort before examining underlying mechanisms. Cognitive impairment, ranging from mild cognitive impairment (MCI) to dementia, represents a significant health concern, particularly among older and frail adults, which impacts their quality of life and places a burden on healthcare systems (3). It is significantly more common in BC patients than in the general population. In a German population-based study of 4,145 subjects aged 50–80 years, the prevalence of mild cognitive impairment (MCI) was 7–12% (4). In contrast, the incidence of MCI according to the Dementia Detection test prior to surgery was observed in 27% of radical cystectomy patients, 80% male, with a median age of 69 years (5), and in 25% of NMIBC patients, the mean age was 78.9 years (6).

In contrast, the incidence of MCI according to the Dementia Detection test prior to surgery was observed in 27% of radical cystectomy patients, 80% male, with a median age of 69 years (5), and in 25% of NMIBC patients, the mean age was 78.9 years (6).

In a series of studies, delirium is a frequent postoperative complication, more often observed in elderly patients and in patients with impaired cognitive status (7). The average age in the study by Lange et al. was 77.8 years (8). Delirium was observed with an incidence rate of 8.0–12% in patients after transurethral resection of bladder tumor (TURBT) (7, 9), and in 29% after radical cystectomy, and was associated with older age, lower preoperative MMSE score, and lower BMI (8, 9). Patients who developed POCD were more likely to undergo reoperation (odds ratio 9.2, *p* = 0.03) and readmission (odds ratio 10.7, *p* = 0.01) (8).

Delirium can be associated with inflammatory markers and may severely compromise outcomes. In the study of Brattinga and coworkers, NGAL and IL-10 were associated with delirium in 311 oncosurgical patients, whereas CRP, IL-1β, and IL-6 were not (10).

In addition to the need for more intensive care during hospital treatment, cognitive impairment can be a reason for non-adherence to prescribed treatment measures, poorer outcomes of surgical treatment, and discharge to a care facility instead of home treatment (11). Therefore, recognition and early intervention could help reduce the negative consequences of cognitive impairment on outcomes. This is especially important because even suspicion of dementia using the Dementia Detection test was predictive for higher perioperative complication rates (29% vs. 5%) (5). These complications in patients with advanced bladder cancer may include anastomotic leak, conduit insufficiency, secondary hemorrhage, subsequent sepsis, myocardial infarction, and even a lethal outcome (5). Understanding the causes and early recognition of new cognitive changes may help prevent further deterioration and guide and improve clinical care, ensuring that mental health is treated alongside physical health (12, 13).

While numerous tools are available to identify cognitive changes in the perioperative period, many challenges remain in clinical practice. One of them is the lack of standardized guidelines for preventing cognitive decline during the perioperative period. Inconsistency in procedures can lead to different patient outcomes and a lack of consensus on best practices for prevention and treatment (14). The International Nomenclature Consensus Working Group recommends that ‘perioperative neurocognitive disorders’ be used as an overarching term for cognitive impairment, postoperative cognitive decline (POCD), and delirium identified both in the preoperative and postoperative period (15).

## 3 Demographic characteristics and baseline risk factors

The population of patients with bladder tumors has some specific characteristics that distinguish them from the population with other tumors, such as colorectal carcinoma. Older age and male gender have been described in a number of studies as risk factors for bladder tumors (6). The male gender is dominant in a series of studies in patients with bladder cancer, where its frequency ranges from 70 to 80% (5, 6, 16), while in colorectal carcinoma, the difference in the incidence of the disease is similar in men and women, and is 53%:47% (17). Other demographic characteristics and risk factors of these populations also differ (Table 1).

**Table 1:** Comparison of demographic characteristics of patients with bladder and colorectal cancer.

Characteristic	Bladder cancer	Colorectal cancer
Age at the first diagnosis	71–73 (16, 18)	68 years (19)
Gender (male: female)	80: 20% (5)	53: 59% male (17, 20)
Risk factors	Smoking, occupational exposure (21)	Diet, obesity, red meat consumption (22), ultra-processed food (23)
Smoking history	57% of patients are smokers (5)	13.7% of the patients are current smokers or stopped smoking in the year of diagnosis (20)
Cognitive impairment	27% (5)	17–24%, *N* = 372 (24)
Comorbidities	Hypertension (56–59.9%). CVD (23.4%). Diabetes (22.4–28%) (25, 26)	No comorbidities: 63.2%: at least one comorbidity: ~33%. CVD alone: 9.4% Diabetes alone: 13.95% Both diabetes and CVD: 4.2% Other comorbidities (COPD, liver disease, kidney disease, etc.): 9.3% (27).
Allergies	Increased risk for BC (RR 1.31) (28).	Decreased risk for CRC, odds ratio 0.64–0.88 (29).
Increased risk	Chronic bladder inflammation (30). Long-term risk: catheterization, diabetes, prior pelvic irradiation (31).	Age, race, and ethnicity dietary habits, obesity, diabetes, previous history of cancer or intestinal polyps, alcohol consumption and smoking, family history of CRC (32).
Survival	Smoking: High risk and strong survival impact for bladder cancer (33). Sex – worse outcomes in female patients (adjusted hazard ratio, 1.24) (34). Worse outcomes of 30,390 new diagnoses in female (hemodialysis 4.6% in men and 18.5% in women) (35).	Smoking increases the risk of CRC by 15–20%. Daily cigarette consumption (RR = 1.38 for >40 cigarettes/day). Duration (RR = 1.20 for >40 years of duration). Pack-years (RR = 1.51 for > 60 pack-years). Age of initiation (RR = 0.96 for a delay of 10 years in smoking initiation) (36).
Disease survival	70% (40–80%) (21, 37). Loss-of-life expectancy in non-muscle-invasive bladder cancer (3.17 years for men vs. 7.14 years for women) (35). 5-year progression-free survival about 70% (38).	73.3% in 2010–19 (39). Age-adjusted 5-year relative survival is higher in women (in Germany, 64.5% vs. 61.9% in men, *p* < 0.0001) (17).

BC, bladder cancer; CRC, colorectal cancer; CVD, cardiovascular disease; RR, risk ratio; COPD, chronic obstructive pulmonary disease.

As shown in Table 1, the greatest risk factors for BC are male gender, a very high smoking rate, and a high incidence of hypertension (5, 25, 40). Both biological and lifestyle risk factors are important, among which exposure to toxins, aniline dyes, solvents, smoking cigars, cigarettes, and other tobacco products are the most studied (41). Occupational carcinogens, such as aromatic amines like benzidine, *α*-naphthylamine, and *β*-naphthylamine, were recognized as directly associated with the development of bladder cancer (42). BC has been experimentally induced in animals after exposure to dyes, and the risk of BC development lags behind years after exposure has ceased (42, 43).

The link between smoking and the development of cancer is the presence of carcinogenic substances in the urine of smokers, including aromatic amines. According to Hecht and coworkers, a total of 83 carcinogenic substances have been confirmed in cigarettes, among which the most significant are powerful tobacco-specific carcinogens, N-nitrosamines, and polycyclic aromatic hydrocarbons (44–46). These carcinogenic substances are absorbed from the lungs into the bloodstream and then reach the bladder via the kidneys. Because urine remains in the bladder longer, the bladder mucosa is exposed to carcinogenic substances. Four variables were found to be associated with cancer development: age of initiation, duration (40 years of duration), daily cigarette consumption, and pack-years (47). In addition to its causal relationship with cancer development, tobacco smoking is a risk factor for the recurrence of bladder tumors as well (43). Carcinogens produced during smoking lead to an increase in markers of systemic inflammation, oxidative stress, and respiratory function disorders measurable by spirometry, which can cause chronic obstructive pulmonary disease (COPD) over time (48). The prevalence of diabetes in patients with BC is also high. It amounts to about 20% and may complicate patients’ treatment (25, 49).

## 4 Etiology and underlying causal mechanisms

The etiology of cognitive disorders in patients with BC is multifactorial, spanning baseline cancer pathology, demographic vulnerabilities, chronic toxic exposure, recurrent infections, systemic toxic treatment burdens, and overlapping psychiatric complications.

### 4.1 Cancer-related cognitive impairment

Cancer-related cognitive impairment may be a first sign of the spread of the malignant disease, but also of the therapies applied. These include slower processing speed, difficulty concentrating and attention to activity, memory lapses, chemo brain, brain fog, and difficulty multitasking (50). Confusion and disorientation, as well as difficulty speaking and writing, were also reported in BC patients (51). This neurocognitive vulnerability serves as a baseline causal mechanism upon which perioperative surgical stressors are superimposed.

### 4.2 Advanced age and frailty

Advanced age and frailty are well-known factors that may contribute to cognitive impairment in the population of urological patients with bladder tumors (6). Multiple comorbidities with different medications are present in this elderly population, with 87.4% of patients having ≥2 chronic conditions (6). Hypertension is strongly associated with changes in microcirculation and smoking (25). Furthermore, long-term exposure to common risk factors for BC development, such as aniline dyes and solvents, increases the risk of cognitive disorders with years of exposure (52). These substances are associated with the development of neurodegeneration and oxidative stress. Therefore, cognitive dysfunction in these patients will progress simultaneously with the development of cancer (52, 53).

### 4.3 Smoking and vascular pathology

Smoking is also one of the risk factors associated with the weakening of a person’s cognitive status (41, 54–56). In addition to confirmed carcinogenicity, exposure to these toxins is significantly associated with cognitive disorders and favors the development of hypertension, cardiovascular diseases, and cerebrovascular diseases (41, 57, 58). The development of cognitive disorders is more common in patients with microcirculation disorders, which is a consequence of smoking (5). Microcirculation damage associated with smoking results in earlier onset of dementia, Alzheimer’s disease, and Alzheimer’s disease-related dementias in smokers compared to non-smokers. Hypertension also leads to arteriolosclerosis, characterized by concentric, fibrotic thickening of penetrating arterioles, often with venous collagenosis and amyloid angiopathy (59). Changes in the microcirculation associated with brain perfusion dysregulation, such as capillary stasis, reduced capillary blood flow, small ischemic foci or microinfarctions, diffuse white matter lesions, and, less commonly, subcortical microhemorrhages, are common in dementia (60). They can also be associated with inflammation, endothelial dysfunction, and hypoperfusion, targeting the anterograde and retrograde cerebral vascular pathway (61).

### 4.4 Infections and systemic inflammation

Even though transurethral resection of bladder tumor (TURBT) is considered a low-risk operation, up to 43% of patients undergoing these interventions have some complications during the first 30 post-operative days, most commonly infections, as presented in Table 1 (26, 62). Multiple instrumental interventions, such as cystoscopy and catheterization, were complicated by hematuria, frequent infections, and exposure to antibiotics and other medications (16). Pyuria, as a complication, has been confirmed in 31.2% of patients with BC, of whom 58% had hematuria, too. These complications are more common in older patients and those with advanced disease (16, 63). Frequent infections in patients with bladder cancer may be related to carcinogenesis. A study by Hu et al. confirmed high levels of N-nitrosamine formation in patients with urinary infections (64). These patients may have 3–12-fold higher concentrations of carcinogenic N-nitrosamines in urine than healthy subjects. Several species, such as *Fusobacterium, Escherichia coli, Klebsiella* spp.*, Proteus mirabilis, Enterobacter* spp., and *Staphylococci*, are thought to act synergistically in BC initiation by forming carcinogenic N-nitrosamines (65, 66). UTI risk is higher in patients with ASA III and IV status and is about 3–10% (67). In the study by Martínez-Delgado et al., positive urine cultures were recorded in 14% of BC patients preoperatively, and in 10% postoperatively (68). The most frequently recorded pathogen was *E. coli* (68). Other bacteria degrading polycyclic aromatic hydrocarbons were typically found in the urine of bladder cancer patients: *Sphingomonas, Acinetobacter, Micrococcus, Pseudomonas,* and *Ralstonia* (69).

The incidence of urinary tract infections is higher in patients with diabetes. Within the group of diabetic patients with BC, those treated with sodium-glucose cotransporter-2 inhibitors (SGLT2) had significantly more infections and longer duration of pyuria compared to diabetics who did not use these drugs (49). Glucosuria in diabetics is probably a consequence of the disease, but can be drug-enhanced, linked to the effect of SGLT2, which further increases glucosuria and thus can favor the growth of bacteria, especially *Fusobacteria*, and *E. coli*, which in turn produce carcinogenic N-nitrosamines (65). In addition to carcinogenesis, urinary tract infections may be associated with delirium, disorientation and other cognitive disorders (70).

### 4.5 Local and systemic treatment protocols

The frequency of systemic chemotherapy among patients with bladder cancer is relatively high because these patients often have other synchronous cancers due to the influence of shared etiological factors. The most common are prostate, lung, or pelvic cancers (71). Most systemic chemotherapy protocols are known to cause cognitive decline. In a study conducted on 135 patients taking tamoxifen for 2 years and the same number of age-matched controls, it was confirmed that the prevalence of cognitive impairment in women taking tamoxifen is significantly higher than in age-matched controls without cancer (47% vs. 28% in the matched control group, *p* = 0.002). This effect was dose-dependent, especially in younger women (72). Moreover, self-reported cognitive difficulties, like processing speed, executive functioning, motor functioning, and verbal learning, are associated with tamoxifen-related side effects, anxiety, depression, and fatigue (*p* < 0.05) (72). Similar effects were observed in the male patients exposed to androgen deprivation therapy, who were 2.10 times more likely to develop cognitive toxic effects than the unexposed or control group (73), AD and dementia (21.6% vs. 15.8%; *p* < 0.001) (74). The most used intravesical chemotherapies are mitomycin C or gemcitabine, usually in combination with intravesical Bacillus Calmette–Guérin (BCG) Immunotherapy (26, 75). To date, no studies have examined whether intravesical mitomycin (MMC) administration influences cognitive status in humans. Preclinical studies investigating the effect of mitomycin on the mouse prefrontal cortex have confirmed that MMC treatment decreased global DNA methylation and increased DNA hydroxymethylation. This effect is strongest in the prefrontal cortex of female mice, mimicking alterations observed during brain aging (76).

**Figure 1:**
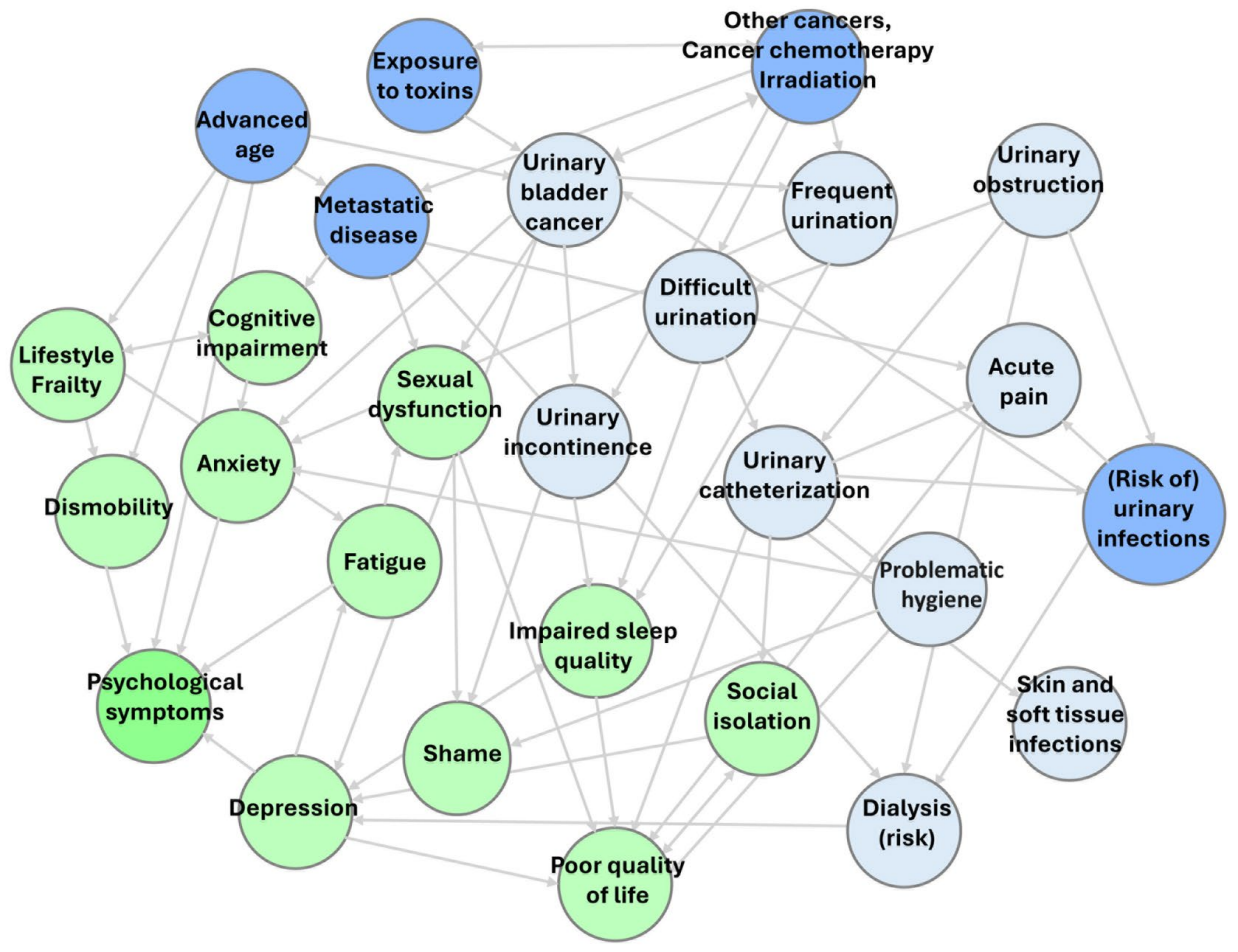
Factors associated with cognitive impairment in bladder cancer patients.

In addition to the cognitive disorder, which is probably caused by microcirculatory changes that occur in the brain under the influence of risk factors, other neurological disorders that can be attributed to chemotherapy were also recorded in these patients, namely fecal urgency, fecal leakage, loss of sexual function and sexual intimacy, and male sexual problems (77). Lower urinary tract symptoms like pelvic pain, severe urinary urgency, and frequency are reported in ~70–80% of patients in the initial 48 h following BCG infusion. These symptoms can be debilitating, especially in complex treatments (38). Withdrawal rate with intravesical BCG therapies is from 20% up to 90% in intensive schedules, due to side effects and treatment burden, particularly during the first year (38, 78, 79) (Figure 1). Previous studies have suggested that patients who received BCG would have better cognitive outcomes and less new-onset dementia during BC treatment (80). However, more recent results from a Swedish nationwide cohort study of 38,934 patients in the NMIBC cohort confirmed only a marginally lower risk of developing Alzheimer’s and other dementias after BCG treatment (81). This benefit was most pronounced among females with advanced age (81).

### 4.6 Associated psychological distress

Psychological factors are often associated with cognitive impairment. Depression and anxiety are diseases of modern civilization, and they have only occasionally been investigated in BC patients, most often in advanced disease and with more aggressive therapies (82). In the cohort consisting of 159 patients, Mohamed et al. registered clinically significant depression in 17% of patients. Borderline abnormal levels for anxiety and depression were reported in another 13 and 17% of the patients (82). A somewhat higher frequency was recorded in the study by Qian et al., which included 104 patients with non-muscle invasive bladder cancer. The authors recorded a frequency of anxiety of 28% and depression of 33%. In their study, anxiety was significantly associated with chronic pain (OR: 3.447) (83). In a systematic review by Thomas and coworkers, which analyzed the factors influencing the increase or decrease of psychological distress in bladder cancer patients, the percentage of psychological distress, depending on the methodology used, varied from 78 to 3.6% (84). Factors that increased psychological distress were advanced pathological/tumor stage, younger age, female sex, and preoperative setting (84, 85). Clinical factors that influenced the reduction of psychological distress were inpatient rehabilitation procedures and the transition from the preoperative to the postoperative period (84).

The prevalence of depression is more common in older patients and those who lack emotional support from loved ones. In a study by Canoui-Poitrine et al. of 1,121 consecutive cancer patients, 14% of whom had bladder cancer, clinically significant depression according to the criteria of the Diagnostic and Statistical Manual of Mental Disorders (4th edition) was reported in 28.4% of patients (86). In this group of patients with a mean age of 80.4 years, depression was independently associated with cognitive impairment (86). Several geriatric assessment findings were independently associated with clinical depression, regardless of gender, metastatic status, and tumor site. These factors were cognitive impairment, multimorbidity, inadequate social support, impaired mobility and function, and polypharmacy (86).

## 5 Perioperative stressors and clinical assessment modalities

### 5.1 Perioperative risks for cognitive dysfunction

Frequent diagnostic and therapeutic interventions can contribute to the development of anemia, thereby increasing the risk of kidney failure, the need for hemodialysis treatment, and increased stress. Newly diagnosed patients with BC are receiving a median of 3.0 cystoscopies per year (87). The average lifetime number of cystoscopies is 6, according to Koo et al. (1), while intravesical BCG can be applied through 27 instillations (38). In addition to intravesical chemotherapy, these interventions, especially at the first appearance of NMIBC, can be the cause of iatrogenic injuries of the urinary tract, the occurrence of hematuria, frequency, urgency, infection, and other lower urinary tract symptoms (88).

Frequent exposure to local or systemic antitumor chemotherapy may be associated with perioperative neurocognitive disorders (26). All these symptoms, together with the constant fear of disease progression, can significantly impair the quality of life in BC patients and cause them additional stress (89).

Although patients with BC are an older population, their HRQOL is significantly worse in all domains compared to the age-matched general population, according to the results of a study by Singer and associates (90). Pain and postoperative distress after TURBT are usually considered insignificant and are most often neglected in the management of patients with NMIBC (16) According to the results of the QOL questionnaire, in which low symptom scores indicate good quality of life, pain in BC patients was 43.8 versus 28.5 in the general population (90).

Enhanced pain perception, particularly suprapubic pain, is more frequently reported among females and patients with advanced disease (stage ≥ bladder cancer) (16). Approximately 30% of these patients experience pain concurrently with bladder spasms, dysuria, and incontinence (16), while nearly two-thirds (64%) report significant procedural discomfort or anxiety (1). Notably, postoperative pain scores are significantly higher following general anesthesia compared to spinal anesthesia (91). Severe pain after radical cystectomy is usually treated, while pain after TURBT is sometimes considered less significant compared to the treatment of BC. It is known that BC patients can have debilitating detrusor spasms, catheter-related pain, bladder discomfort, fatigue, and mental health issues (92). This pain is moderate to severe in 27.3% of patients (93) whereas significant discomfort was reported in up to 60% of patients (94). Inadequately managed, this continuous visceral pain serves as a potent trigger for patient dissatisfaction, the neuroendocrine stress cascade, sleep disruption, directly accelerating the onset of postoperative delirium (95).

Beyond clinical characteristics, pain perception in bladder cancer patients is also influenced by demographic and psychosocial factors, such as gender and marital status (96). In an evaluation of 252 male patients undergoing treatment for non-muscle-invasive bladder cancer (NMIBC), Krajewski et al. demonstrated that pain tolerance was significantly higher in patients with partner support. Conversely, single patients experienced significantly higher rates of pain catastrophizing, anxious preoccupation, and feelings of helplessness (96).

Although preoperative anemia in BC patients is associated with poorer oncological outcomes—such as cancer recurrence and lower overall survival—data regarding its connection to adverse cognitive outcomes remains lacking (97). Instead, cognitive risks in these patients are frequently tied to surgery; in elective open urological procedures, intraoperative bleeding, hypotension, and transfusions are major risk factors for delirium, compounded by preoperative COPD and alcohol use (95). These intraoperative complications cause cerebral hypoperfusion and hypoxia, which are well-established triggers for the neuroinflammatory cascade. These risks may be effectively reduced by opting for minimally invasive surgery (98).

#### 5.1.1 Anesthesia and cognitive outcomes

The use of certain drugs may be associated with the occurrence of delirium. It was confirmed that the frequency of dementia and delirium is more common in BC patients who preoperatively used benzodiazepines (7).

There were implications for a higher incidence of POCD related to a certain type of anesthesia or medications used during anesthesia. Currently, hypotheses regarding how anesthetic techniques affect cognitive outcomes in BC patients are largely extrapolated from broader oncologic and geriatric surgical populations, where propofol-based anesthesia and regional techniques have shown potential neurocognitive benefits (99). Intraoperative drug choices also play a critical role. A significantly increased risk of delirium was registered in surgical patients administered non-depolarizing neuromuscular blockers without a reversal agent compared to those who received a reversal agent (OR 1.52, *p* < 0.001), with no difference observed between the administration of neostigmine or sugammadex (100).

Certain adjunctive therapies may show protective potential. A meta-analysis pooling data from 1,626 adults aged 60 or older undergoing general surgery demonstrated that dexmedetomidine significantly reduced the incidence of POCD, as assessed by the Mini-Mental State Examination (MMSE) (risk ratio: 0.47, *p* < 0.001). However, the baseline anesthetic regimens in these trials were not always clearly specified, as the included studies primarily compared general anesthesia protocols with or without dexmedetomidine (101). Furthermore, alternative strategies like opioid-free anesthesia—utilizing a combination of dexmedetomidine, ketamine, and lidocaine—showed no statistically significant changes in postoperative MMSE scores, though this was in a small pilot sample of 15 patients undergoing minor urological procedures (102).

The only anesthesia-related predictor of postoperative neurocognitive decline was longer duration of anesthesia, which in turn reflects complexity of surgery, and not a type of anesthesia (103). On the contrary, preoperative mild dementia, impaired cognition, comorbidities, older age, and lower body mass index in urological patients were consistently associated with postoperative delirium, transient or persistent cognitive deterioration, and postoperative complications (5, 9, 104, 105).

Postoperative quality of recovery following TURBT was found to be better in patients anesthetized with sevoflurane in comparison to those anesthetized with remimazolam, especially in the domains of psychological dimension and pain control (106). In a study by Kim et al., which included 488 urological patients, it was confirmed that postanesthetic emergence agitation was observed in patients with severe pain, previous alcohol consumption as well as catheter-related bladder discomfort (107).

The type of anesthesia may influence bladder cancer outcomes. In a study of 287 patients with NMIBC, recurrence and progression rates were higher in those receiving general anesthesia (39.8% vs. 27.4 and 15.2% vs. 7.8%, respectively) than in those receiving spinal anesthesia (108). Similarly, a meta-analysis found that regional anesthesia was associated with a lower risk of tumor recurrence (HR 0.55, 95% CI 0.39–0.79; *p* = 0.001) and improved 5-year survival compared with general anesthesia (109). However, there are no well-controlled randomized studies to confirm this observation in BC patients. Recent meta-analysis did not confirm a clear difference between specific anesthesia techniques and their association with cognitive outcomes in adult surgical patients (110). Likewise, no significant differences in long-term survival have been demonstrated between patients receiving propofol-based total intravenous anesthesia and those receiving sevoflurane-based general anesthesia during BC surgery (111).

### 5.2 Standard cognitive assessment modalities

Preoperative cognitive screening is essential when evaluating urological patients, particularly to identify candidates for extensive surgical procedures or therapies such as radical cystectomy and pelvic irradiation (5, 112). Various tools are used to conduct these assessments effectively, most notably the MMSE. The MMSE evaluates core cognitive functions—including orientation, memory, attention, language, and visuospatial skills—and serves as a rapid, effective tool for detecting Alzheimer’s disease and dementia (87–91% specificity, 77–83% sensitivity) (113). However, its utility is limited when screening for mild cognitive impairment, where its sensitivity ranges from 61 to 66% and specificity from 65 to 74% (113).

In a group of 51 urological patients, MMSE and Dementia Detection Test (DemTect) were compared. DemTect identified suspected mild cognitive impairment in 27% (14/51) of participants, whereas the MMSE and clock-drawing test detected abnormalities in only 10 and 6 patients, respectively. Notably, only 25% (5/20) of patients with positive DemTect results were deemed suitable for a continent neobladder diversion by their physician. Furthermore, a suspected DemTect result was associated with significantly higher perioperative complication rates (29% vs. 5%) (5).

Another commonly used tool is the Montreal Cognitive Assessment (MoCA), originally developed to detect mild cognitive impairment and also used as a screening test for dementia. MoCA is more sensitive to mild cognitive deficits than MMSE, with a sensitivity of 79–90 and a specificity of 70–81%, whilst it has a sensitivity of 91% and a specificity of 81% for AD/dementia (113). The MoCA provides a comprehensive evaluation of several different cognitive domains: attention and concentration, executive functions, memory, language, visuoconstructional abilities, conceptual thinking, calculation, and orientation. The total possible test score is thirty points, and a score of twenty-six points or more is considered normal (114).

The Mini-Cognitive Assessment Instrument (Mini-Cog) is a validated screening tool for cognitive impairment, demonstrating 91% sensitivity and 86% specificity for detecting dementia. Scored out of a maximum of 5 points, the test consists of two components: a clock-drawing test and a delayed 3-word recall task (7). In a study of 198 patients undergoing TURBT, a Mini-Cog score of less than 3 predicted probable cognitive impairment in 19% of patients and served as an independent risk factor for postoperative delirium (7).

Some scales are particularly applicable to cancer patients, such as the Mini-Mental Adjustment to Cancer (Mini-MAC), the Acceptance of Illness Scale (AIS), and the Coping Strategies Questionnaire (CSQ) (96). In addition to confirming dementia, these tests may assess the patient’s psychological state, and the resulting data can be used to evaluate the ability to accept complex treatment protocols.

Clinical frailty is increasingly investigated in the perioperative assessment of patients. Several scales have been used to screen for frailty in the perioperative period. One of them is the Clinical Frailty Scale (CFS). The CFS rates patients from one (meaning very capable) to nine (representing a terminally ill patient), based on their daily functioning and the impact of chronic health conditions on their daily activities (115). The importance of clinical frailty in the perioperative evaluation is increasingly highlighted, as it has been observed that as many as 43% of NMIBC patients are prefrail or frail (6, 116).

### 5.3 Preoperative intrinsic capacity and circulating biomarkers

An assessment of preoperative intrinsic mental and physical capacity in bladder cancer patients aims to identify high-risk patients by deficits in cognition, mobility, and vitality who are candidates for targeted prehabilitation (117). Mini-MAC analyses methods of cancer coping, utilizing a 29-item questionnaire that distinguishes patients with a positive spirit from those with a destructive coping style (96). Using the 4-item Physical Resilience Instrument for Older Adults, Liu et al. confirmed that over 60% of older patients had moderate or low capacity (117, 118). Low mobility and low cognition phenotypes were confirmed to have a significantly increased risk of adverse recovery trajectories (117).

Laboratory assessment of biomarkers of neurological damage is rarely performed in these patients, because diagnostic procedures focus primarily on detecting malignancy (119, 120). Most data regarding circulating neuro-inflammatory markers come from major non-cardiac and cardiac surgical studies (121). The biomarkers examined were primarily pro-inflammatory cytokines, including tumor necrosis factor-*α*, interleukin-1β, interleukin-6, and interleukin-8, as well as biomarkers of neuronal injury, such as S100B and C-reactive protein. It has been shown that reduced cholinesterase activity in patients with delirium was associated with elevated levels of C-reactive protein and IL-6 (122). C-reactive protein (CRP) itself is a biomarker of inflammation, infection, and tissue damage, but it is also associated with cognitive dysfunction in elderly patients (89, 123). S100B is a biomarker of direct neuronal injury, and its association with cognitive changes in the perioperative period has been demonstrated (124, 125).

## 6 Strategies for cognitive impairment prevention

By understanding risk factors for developing cognitive impairment, healthcare providers can implement strategies to help identify patients at risk and promote recovery, thereby improving overall outcomes for patients undergoing urological surgery (105, 112).

### 6.1 Lifestyle and psychological interventions

The treatment outcomes of these patients have significantly improved with the introduction of new treatment methods and the reduction of the incidence of smoking in the general population. Quitting smoking can be a significant factor in preventing the recurrence of the disease and deterioration of cognitive status. Quitting smoking can reduce markers of systemic inflammation and improve muscle fatigue resistance, already 14 days after cessation, thereby minimizing postoperative complications (48, 126). Preoperative counseling about recurrence contributes substantially to patients’ anxiety and worry. Negative psychological outcomes in bladder cancer can be reduced by using support services to alleviate symptoms (127). In a large study by Bahlburg et al., early inpatient rehabilitation significantly reduced psychological distress in patients with radical cystectomy and urinary diversion, which was particularly important in younger women (85). Lack of sleep may lead to chronic fatigue and psychological distress, with insomnia registered in the postoperative period by 19.7% of patients (16). With partner and family support, music was confirmed to be beneficial, too (127, 128). Raheem et al. confirmed that music during flexible endoscopy may reduce anxiety and pain (*p* < 0.0001) (128).

### 6.2 Clinical pre-habilitation and medical optimization

Earlier diagnosis and treatment of urinary tract infections, and possibly bacterial colonization, may decrease the incidence of delirium, alleviate local complications, and reduce systemic effects of infection (64, 70). Results of a recent meta-analysis by Paterson et al. of 1,034 BC patients confirmed that clinical outcomes improved significantly following the application of multimodal pre-habilitation programs (126). Educational programs improved patients’ reported outcomes, whereas exercise-only interventions improved physical function. Supplemental clinical nutrition with high-protein and high-energy intake should be initiated as early as possible during preoperative preparation, especially in malnourished, anemic, or hypoproteinemic patients. Increasing leisure-time physical activity after diagnosis was similarly associated with lower progression risk and improved health-related quality of life (129).

### 6.3 Future horizons in non-invasive diagnostics

To directly reduce the cumulative procedural stress and anxiety driven by surveillance regimens, transitioning to modern molecular diagnostic alternatives is highly recommended. The number of invasive cystoscopies could be reduced with newer diagnostic methods from blood or urine, such as uTERTpm ddPCR, thereby maximizing intervals between interventions and reducing systemic exposure to local and general anesthetics (87, 120, 130). Early stratification of perioperative procedures based on molecular features is highly recommended to minimize overall patient distress and iatrogenic complications (1, 40, 131).

## 7 Conclusion

Cognitive function plays a key role in various aspects of urological care, from surgical planning to device management and pharmacological treatments. Given the complexities of cognitive impairment in urology, healthcare professionals must incorporate cognitive assessments into routine care to enhance patient management and outcomes. Early postoperative symptom care may improve the quality of recovery of urological patients after TURBT.
